# The humanitarian border as a violence‐producing environment: revisiting aid and anti‐migration protests on Lesvos, Greece

**DOI:** 10.1111/disa.12679

**Published:** 2025-02-27

**Authors:** Bram J. Jansen

**Affiliations:** ^1^ Wageningen University The Netherlands

**Keywords:** anti‐migration mobilisation, humanitarian aid, humanitarian border, Mediterranean migration crisis, volunteer humanitarianism

## Abstract

Violent clashes erupted on the Greek island of Lesvos in early 2020, during which aid groups, volunteers, and activists were threatened and attacked. Aid actors and media sources attributed these events to far‐right, nationalist, and xenophobic mobilisation; however, this risks ignoring more structural factors and local perspectives on asylum policies and practices. This paper suggests that a more critical approach is necessary to understand why people mobilised against aid on Lesvos, and it explores how this antagonism can be seen as intrinsic to the ‘humanitarian border’ as it materialised on the island. Aid groups, volunteers, and activists became integral to this, spawning stories of how they were sustaining the migration dynamic. How these stories coincided with far‐right mobilisation is not straightforward, and nuancing how local concern and protest and far‐right anti‐immigration sympathies relate is imperative to comprehending hostility to aid groups and may contribute to fostering better relations with communities in refugee‐hosting areas.

## 1 INTRODUCTION

In the wake of the Mediterranean ‘migration crisis’ of 2015, the Moria refugee camp on the Greek island of Lesvos became emblematic of the migration approaches of the European Union (EU) and the Greek state. Moria camp embodied a humanitarian crisis on European soil, in which more than 15,000 asylum seekers lived in squalor, assisted by hundreds of aid groups, volunteers, and activists from Greece, other parts of Europe, and beyond. Researchers have analysed extensively the conditions in these ‘hotspots’—the first reception facilities in Greece and Italy that came into being as a result of the European Agenda on Migration in 2015—and in particular in Moria camp, focusing on broader migration dynamics, border processes, and the practical effects on migrant reception (Kalir and Rozakou, 2016; Pallister‐Wilkins, 2020; Topak, 2020; Iliadou, 2021; Vallianatou, 2023), as well as on the challenges to humanitarian action, refugee assistance, and human and refugee rights (Papada et al., 2020; Gordon and Larsen, 2021; Witcher, 2022), and on humanitarian volunteerism (Guribye and Mydland, 2018; Knott, 2018; Tsartas et al., 2020). What has been studied much less, though, is how local host communities perceived of asylum policies and practices in these areas, and the impacts on their lives and environment.

In this paper I explore this issue by analysing an episode of violent escalation in which aid groups, volunteers, and activists were threatened and attacked. I argue that the hotspot architecture placed aid groups, volunteers, and activists in roles that were integral to problematic migration management. This fed a discursive framework of neglect of local concerns, which aligned well with nationalist and far‐right discourses that amplified and legitimised anti‐migration sentiment, and which tipped over into protest and violence against aid groups. This relation with the far right is not straightforward, however, and uncritically associating local concerns and far‐right political ideologies risks ignoring more structural underlying causes of protest. Anti‐asylum sentiments are a challenge for aid actors, and failure to tackle and engage with them risks people being drawn to actors with anti‐humanitarian ideologies, who are seen as responsive and attentive to these matters. The relation between local concerns and experiences of neglect and far‐right attitudes and xenophobia is complicated and how the two coincide in escalation is nuanced. This paper contributes to studies on the dynamics of humanitarian borders and anti‐migration protests and mobilisation against aid in such settings.

The paper is based on qualitative fieldwork and interviews with aid actors, activists, and local people in 2021, as a follow up to earlier research on violence and protest in the Greek hotspots in 2020,^1^ in addition to a desk study of academic, policy, and (social) media resources. The fieldwork was conducted from 1–8 July 2021 and consisted of 23 interviews with members of local aid groups, international non‐governmental organisation (NGO) workers, volunteers, business owners, local activists, and a journalist, as well as focus‐group discussions and interviews with local community members in various locations on Lesvos, including Moria, Mytilene, Panagiouda, and Paralia Thermis.^2^ In addition, data were used from several online interviews from an earlier study in summer 2020, also conducted by the author (Jansen, Weishaupt, and Lubberdink, 2020), and supplementary data were utilised from fieldwork performed on Lesvos by a research assistant in September 2020. Interviews lasted between 60 and 90 minutes on average and were not recorded. Names and affiliations are anonymised at the request of most participants, but places, roles, and dates are given in the endnotes.

## 2 THE HOTSPOT ARCHITECTURE AS A VIOLENCE PRODUCING ENVIRONMENT

The Greek island of Lesvos, located roughly eight miles from the Turkish coast in the Aegean Sea, became a popular point of entry into the EU for asylum seekers fleeing Afghanistan, Syria, and other counties. From around the mid‐2000s, mainly Iraqi refugees found their way across the ‘blue border’ (Papataxiarchis, 2016a) and since then, the Aegean Sea has become a regular scene of population movement. In 2015, at the height of the ‘the long summer of migration’ (Witcher, 2022), almost 700,000 people moved through the island (Papada et al., 2020). The surge in arrivals led to the creation of the ‘migration hotspots’. These ‘first reception facilities’ were a component of the approach presented by the European Commission as part of the European Agenda on Migration in 2015, and they sought to improve the coordination of EU agencies' and national authorities' efforts during the initial reception, identification, registration, and fingerprinting of asylum seekers and migrants. The hotspots were located in Greece and Italy, as frontline states of European migration. In Greece, hotspots on the islands of Chios, Kos, Leros, Lesvos, and Samos were designated for assisting asylum seekers into asylum procedures and implementing relocation schemes and return operations (Luyten and Orav, 2020).

Lesvos as well as other islands in the Aegean Sea became what Vives (2017, p. 191), referring to Cuttitta, describes as a ‘spectacular border’, in which the plight of refugees, and the effects of the processes of managing the arrivals, such as encampment, incarceration, and pushbacks, became visible. The spectacular border has a two‐sided nature, encompassing not only the threatening, militarised, and law‐enforcement aspects of cross‐border mobility, but also its humanitarian features (Franko, 2021, p. 380). In effect, the two sides become intertwined in a ‘humanitarian border’, a conceptualisation of ‘zones of humanitarian government along the territorial edges of nation‐states’ (Williams, 2015, p. 11). These are spaces where border crossing is a matter of life and death (Walters, 2011) and where rescue measures meet those of containment and deterrence (Dijstelbloem and van der Veer, 2021). As a result of Greek and EU securitisation policies and their racialised deterrence, in themselves a form of border violence, asylum seekers and migrants had to undertake dangerous sea crossings in their attempts to access Greece.

In response to the increase in arrivals in the EU hotspots in Greece, the *EU–Turkey Statement* of 18 March 2016 stipulated that asylum seekers who were deemed ineligible for protection would be received by Turkey.^3^ For each asylum seeker sent back, another one found to be eligible would be resettled, moving from Turkey to an EU member state. In exchange for financial support, the Turkish authorities were to prevent irregular migration from the shores of Turkey to the Greek islands. After the implementation of the Statement, the number of new arrivals decreased significantly, yet the redistribution of asylum seekers within the EU stalled and hence the Greek hotspots began to function as spaces of containment and immobility (Topak, 2020; Vallianatou, 2023). Migrants became stuck on hotspot islands such as Lesvos, most notably in and around Moria camp (Kalir and Rozakou, 2016). The latter became a ‘bottleneck’ (Gordon and Larsen, 2021), and Greece was ‘ring‐fenced’ and largely left alone in taking care of the thousands of migrants on its shores and islands (Hilhorst, Hagan, and Quinn, 2021).

The hotspot approach as it enveloped Lesvos can be seen as a manufactured emergency (Papada et al., 2020). By preventing onward movement to the mainland, Lesvos became a containment site where the number of people surpassed available facilities: Moria camp was designed for 3,000 people, yet it hosted around 15,000 asylum seekers in late 2019. The implication was akin to a consolidation of crisis (Pallister‐Wilkins, 2020): asylum seekers who got stuck on the island settled in and around Moria and several other places in dire circumstances that grew worse over time in terms of hygiene, shelter, health, safety, and nutrition (Reidy, 2019).

Given the increase in arrivals on Lesvos, aid workers, volunteers, and activists came to the island to assist with the reception of asylum seekers, in addition to the many local people who started grassroots aid initiatives. Some people came with established international NGOs such as the International Rescue Committee, Médecins Sans Frontières, and the Red Cross, and multilateral organisations such as the International Organization for Migration (IOM) and the United Nations Refugee Agency, but also many spontaneous endeavours emerged. Some were based on local engagement, others involved international volunteer groups, or were collaborations between the two. Eventually there were hundreds of different initiatives and groups doing something humanitarian‐like on Lesvos (Nianias, 2016; Guribye and Mydland, 2018; Knott, 2018). Pallister‐Wilkins (2022, p. 139) referred to Lesvos in those days as a ‘humanitarianesque carnival’ in which the number of grassroots volunteers increased after every ‘delicious horror’ report. In a similar vein, Papataxiarchis (2016a, p. 6), an anthropologist who witnessed some of these events, notes how ‘the very sight of an inflatable refugee boat creates a burst of energy – some have called it “a festival” (*panigyri*) – among the volunteers’.

The crisis as it unfolded on these shores, as well as its mediatisation and location, contributed to an appeal for people to engage with, regardless of, or perhaps because of, Greek policy and intervention. Lesvos became a cause, and it seemed it was almost celebrated by hundreds of young people who came to the island and settled in Mythimna, Mytilene, or Skala Sikamineas, as part of organised volunteer tours ranging from two weeks to a couple of months. Many of them were inexperienced or had been unexposed to aid work, or the broader European and Greek asylum dynamics and the role and position of the Greek state or humanitarian and aid groups, or the wider context of Lesvos in relation to these aspects.^4^

Reverberating such dynamics, borders and border processes can be seen as ‘theatres of sovereignty’ (Franko, 2021). They are the scene of interplay among states, migrants, and governmental and non‐governmental organisations and activists that have become engaged in the ‘design, policing, administration, and legal and technical operation of the border’ (Andrijasevic and Walters, 2010, p. 978). In this reading, the presence of so many volunteers, activists, and NGOs can be understood as a form of ‘gap filling’, meaning that where governments fail to provide protection, a needs gap materialises that is filled by these aid groups (Cuttitta, 2023). Indeed, aid groups were supplementing or even taking over the role of asylum seeker reception and the search and rescue activities of the state and Frontex, the European border and coastguard agency, in the Mediterranean. As such, humanitarians, volunteers, and activists became engaged in deciding on matters of life and death, a prime sovereign domain (Franko, 2021, p. 382).

The EU hotspot environment is an exemplary example of a bordering process that reveals competing ideas of territorial sovereignty. Humanitarians, activists, and volunteers ignore, neglect, or confront state and government policies in their engagement with asylum, and are in this sense symbols against violent border practices. Sandri (2018) argues that the volunteer engagement enabling the ‘Jungle’ migrant settlement of Calais can be understood as civil disobedience in defiance of the French state. Similar associations were made by volunteers I met and who saw the activism and assistance to asylum seekers as negating a distant, unwilling, and racist government/EU. Yet, in the absence of a comprehensive government response, aid groups and NGOs came to cater for asylum seekers themselves. To varying degrees, they became integral to the migration management practices in places where people get stuck or remain incarcerated, a process indicating the ‘NGO‐ification’ of EU migration management (Cuttitta, 2015, 2023). Rather than as symbols in defiance of the state, this reading shows NGOs as complementary to or extensions of the state.

Aid groups on the island were not a uniform body of actors. Multilateral organisations such as the EU, the IOM, and the United Nations, NGOs, as well as activist and local or individual initiatives, or volunteers, all had diverse agendas and practices. This variety was significant as it shaped the political dynamics around asylum in the hotspot. Many activists went by the label ‘solidarians’, engaging in more informal, anti‐institutional, and anti‐hierarchical aid (Rozakou, 2016; Fotaki, 2022), and seeking to disengage from the more established and formal NGOs, volunteer organisations, and the government. And established NGOs sought to distinguish themselves from each other, such as in relation to encampment and exclusion policies.

The intricacies of these differences may, however, only be visible to insiders of ‘Aidland’ (Apthorpe, 2011) and be largely invisible to host communities. Or irrelevant. Perhaps with the exception of local grassroots movements, international aid groups can be seen as a related body of actors that came to co‐shape the conditions of asylum reception on the island, and as such, were more of a collective or community than they imagined themselves. This points to a ‘perception gap’ (Donini, 2007) between aid givers and societies. Divisions and reputations among aid groups, and the positionality and humanitarian identity of agencies, may matter much less than the humanitarians themselves hold on to. In this sense, aid workers and solidarians on Lesvos may perceive their involvement in the hotspots in radically different ways than the general public.

This raises important questions about the nature of humanitarianism in these border areas. Aid and activism may have unintended consequences and anti‐humanitarian effects. Others have written about ‘humanitarian complicity’ in contributing to long‐term refugee encampment (Chkam, 2016; Papada et al., 2020), ‘humanitarian impunity’ in sustaining violent government actions (Branch, 2008), or maintaining a ‘humanitarian present’ (Weizman, 2011, p. 4) as calculated minimum humanitarian care that keeps further crisis at bay, but is thought to serve a function in deterrence or control. In the case of the humanitarian border along Southern Europe, humanitarian action has been analysed from the standpoint of sustaining a policy of ‘violent inaction’ (Gordon and Larsen, 2021), or as implicitly aiding ‘thanatopolitical border regimes’ (Iliadou, 2021, p. 206). Big words indeed, and they express existential dilemmas that were also perceived by aid actors whose aspirations were challenged by political realities. Yet, for most of the people with whom I spoke, these dilemmas found resolution in something akin to a humanitarian imperative—to relieve suffering wherever it may be found—that drove a responsibility to assist vulnerable people. In other words, the bigger picture was lost to a more urgent and short‐term response, in which humanitarianism can be seen as ‘conflicted care’ (Crane and Lawson, 2020).

The confinement and encampment of so many people in the hotspots culminated in what an NGO staff member referred to as a ‘violence‐producing environment’.^5^ It is well documented how the detrimental situation in these areas shaped conditions of exhaustion and uncertainty, which increased frustration and anxiety and compounded fighting and criminality among asylum seekers and others (Pallister‐Wilkins, 2017; Ansems de Vries and Guild, 2019; Topak, 2020; Aikins, 2022). Yet, the frustration and anxiety of local people and how this fed hostility to aid groups, activists, and associated people, was another matter, and is much less studied. But this tells an important story about the humanitarian border, how aid groups, volunteers, and activists relate to its dynamics, and its effects on local populations. In the run up to the riots of early 2020, an image was produced of volunteers, solidarians, and aid groups on the island as complicit in the migration drama on Lesvos. This emerged in a context in which aid actors were so engaged with the asylum seekers' plight and condition that local discontent went unnoticed or was seen as an aberration or excessive.

## 3 PROTEST, ESCALATION, AND MOBILISATION

In spring 2020, protests on Lesvos escalated into violence against aid groups, journalists, and other actors associated with assisting asylum seekers. According to some respondents, it was the nationalist mobilisation of youth by Greek and international far‐right activists that instigated the riots. For others, it was a long time coming: islanders were responding to an asylum situation that was managed badly, culminating in broader protests against the building of migrant detention centres on the island. During the escalation, aid groups and their staff experienced threats and attacks unknown and unexpected in the European aid context.

The Greek government had recently announced a decision to create permanent migrant detention centres in the hotspots (Schmitz, 2020). For many among the population of Lesvos, as well as aid groups and activists, this was seen as solidifying the island as a permanent migration site, as ‘a refugee warehouse’ without them having a say in this matter (Vallianatou, 2023). This would consolidate the problems they had seen developing since 2015, such as the proposed fencing of the ‘Olive Grove’ and ‘the Jungle’, informal extensions of the overcrowded Moria camp on local farmland. In response, people reacted by protesting against the government and the police force sent from the mainland, but they also turned against those who were seen as enabling this consolidation: aid groups and volunteer humanitarians (Jansen, Weishaupt, and Lubberdink, 2020; Schmitz, 2020). NGOs and volunteers bore the brunt of the protests, and were seen by rioters as facilitating migration and incarceration.

Although tensions were not new on the island, this time the escalation was different. A significant moment, or ‘catalytic moment’ as it is referred to in an anonymous chronology of events,^6^ came when dissatisfied locals—‘vigilantes’ in the document—from Moria village sprang into action. As the story goes, on 3 February 2020, hundreds of refugees were protesting in Mytilene (the capital of Lesvos) against the conditions of their entrapment on the island. Police and the authorities repelled them and directed them back to the camp through Moria. Then the church bells started ringing, to call for the locals to block the refugees. That night, ‘vigilante groups were conducting attacks wearing full face masks, hoodies and carting bats’.^7^ They were looking for refugees, migrants, and foreigners, as well as volunteers and aid staff. Villagers erected roadblocks and checkpoints, where they stopped cars and prohibited refugees and NGO workers from accessing the village. From then until the end of March, threats against and attacks on aid staff, solidarians, and volunteers, and their infrastructure, occurred on a regular basis until many aid groups decided to evacuate the island. Several people I interviewed shared their personal experiences of being threatened, beaten, held, and abused. Other sources also report that NGO cars and other properties were attacked and demolished, a school on NGO grounds was set on fire, and other instances of intimidation by local vigilantes ensued (Fallon, 2020; Jansen, Weishaupt, and Lubberdink, 2020; Maddox, 2020).^8^

This was an escalation that took many by surprise. Aid groups and volunteers had experienced tension and threats before, but the way islanders were mobilised into action this time was different. Even local employees and staff of NGOs were reported to have joined the action.^9^ No one I spoke with from among the aid groups and activists had anticipated that events would turn so violent. They were used to nationalist protests, but how these collided with local resentment, and how they were triggered, was something else. What started as a protest against migrants and migrant management escalated into violence against what was seen as the support structure enabling the asylum seekers' presence on the island: aid groups and activists.^10^

The anti‐immigration mobilisation literature is roughly divided into two main perspectives. The first concerns ‘grievances theory’, concentrating on the material or cultural criticisms that drive mobilisation for protest. The second engages with ‘political opportunity structures’ (POS) as a mechanism driving mobilisation. Here, institutional decision‐making processes and discursive opportunities (that is, media attention) shape mobilisation processes (Segers, 2021, pp. 50–51). Whereas POS theory depends on understanding opportunities for mobilisation, grievances theory zooms in on the internal processes of the making and framing of meaning. Koopmans and Olzak (2004) suggest bridging the two theoretical positions, proposing a focus on ‘discursive opportunities’ in which political opportunity and framing intersect in mobilisation for collective action. Here, political opportunities and framing meet in discursive opportunities where ‘the public sphere mediates between political opportunity structures and movement action’ (Koopmans and Olzak, 2004, p. 201). How certain claims, stories, and events become visible, and find resonance and legitimacy, contributes to people's willingness to rise up. In particular, as I will show below, the role of ‘established political actors who express support for a social movement's actions or demands’ in person and on social media, referred to as consonance, adds to social action (Koopmans and Olzak, 2004, p. 201). This fits with the suggestion that far‐right or nationalist influence affected or exacerbated the rioting; however, rioters turning on aid groups was the result of a build‐up of events in a context in which violence and rioting had already occurred, or rather, had been present for years already, but not to such an extent (Maddox, 2020).^11^

In the lead‐up to the escalation, many political orientations and sectors were represented in protests against the building of closed migration centres: firefighters, police, communists, members of the right‐wing Golden Dawn party, aid groups, volunteers, and solidarians—they all protested against the island becoming a migrant prison (Schmitz, 2020). Yet, this momentum also set the stage for aggression against aid groups and legitimised it to such an extent that local police did not intervene when it occurred (Gordon and Larsen, 2021). In fact, the burning of a refugee school in a NGO compound was applauded on Facebook by a member of the ruling political party (Tselepi, 2020). Such resonance, or consonance, amplifies and legitimises these actions.

The question remains how these events can be seen as social movement action owing to a deliberate campaign, or as a more spontaneous outburst and ad hoc organisation—or something that contains elements of both. The church bells ringing as a call to arms by the villagers of Moria, as well as mobilisation via Facebook and WhatsApp groups, did not occur in a political void. This took place in an already protestive environment. Some days prior to the turn to aid actors, strikes were organised by the municipality of Lesvos and the regional administration of the northern Aegean—attended by thousands of Greeks—under the slogan ‘we want our islands back, we want our lives back’ (Maddox, 2020; Zander, 2020). The legacy of the status quo, more than 15,000 people stuck on the island in early 2020, and lingering stories concerning the role of aid groups created a tipping point.

Ives and Lewis (2019) distinguish between campaign‐level escalation and protest event‐level escalation. The former denotes the mobilisation of people into (violent) action as a result of a political campaign, whereas the latter implies a much more spontaneous eruption of action, as events take place in much more fluid and organic ways (Ives and Lewis, 2019). In this reading, spontaneous organisation of protest, and thus a more horizontal structure of hierarchy, or lack thereof, allows for violence‐oriented actors to break group norms and resort to violent escalation, because the ability of gatekeepers to control protest is diminished. Discursive opportunities play a significant role here, and consonance and legitimacy accorded by leaders and influential insiders and outsiders can add fuel to the fire.

In addition, more spontaneous escalation needs a story, an underlying rationale, what Snow and Moss (2014, p. 1133) describe as ‘behavioral/emotional priming and framing’. They suggest that spontaneous action is not so much random but scripted by prior priming experiences. They define priming as ‘an increased sensitivity to certain stimuli due to prior experiences. It is a pre‐sensitizing process that increases the probability of activating a concept, frame, emotion, or line of action based on exposure to an earlier similar stimulus of experience’ (Snow and Moss, 2014, p. 1134). The relevance of this is that the underlying framing of aid groups, as part of the problematic situation on the island, was a component of the stories and experiences waiting to be triggered.

Mobilisation needs a story. Shared stories shape mobilisation. Furthermore, they ‘knit a diverse group of individuals together in the pursuit of a collective goal’ (Segers, 2021, p. 51), and indeed, contribute to a ‘symbolic perception of crisis, threat, vulnerability, injustice and so forth’ (Koopmans, 2004, cited in Rucht, 2018, p. 227) that motivates and legitimises people's protests. On Lesvos, such a local story concerned how aid workers, volunteers, and humanitarians were seen as enablers of the ‘sacrifice of Lesvos’, as an interviewee termed it.^12^ Aid groups were not neutral in the eyes of some of my respondents; rather, they were part and parcel of the hotspot dynamic that they believed had become untenable, because of, not despite, their perceived role in sustaining it, regardless of the internal diversification in ‘Aidland’ and the positionality that individual groups held on to or thought to espouse.

Yet, among actors directly operating in these environments, such as volunteers, especially those on short‐term missions, there seemed little awareness of their role in the stories. More regular aid staff did reflect in my interviews on how they had become integral to the hotspot dynamic, but less so on how they were seen as such by local people, and how experiences, gossip, and accusations transpired on the island.^13^ People on Lesvos told me about how aid groups were actively supporting crossings from Turkey. On separate occasions people narrated how they believed in aid workers' cooperation with smugglers, identifying routes and facilitating points of entry. As one interviewee remarked: ‘NGOs had an interest, they were guiding people from Turkey, with lights from the coastline to guide boats to landing sites’.^14^ In this understanding, a plethora of NGOs, activist groups, and volunteers were seen not only as aiding people in need, with more conventional aid, food, shelter, and medicines, but also as enabling their mobility and arrival. In this view, aid groups were understood to be in direct contact with smugglers from Turkey, alerting them about where and when to send ships, or where to find appropriate landing points. Similar ideas have also been reported in other hotspots (see, for example, Murray, 2017; Hayden, 2022).

Interviewees and reports suggested that NGOs had informed asylum seekers and assisted them with protesting (Fallon, 2020).^15^ Local media were asking: ‘who taught these Afghan women to write their banners?’.^16^ Given the earlier protests in which asylum seekers and solidarians acted jointly, this question resonated.^17^ Moreover, similar voices were heard around the large protest to which the escalation in Moria was a response. In the same way, an earlier study notes that parts of the local community were aggravated when at some point refugees were going into Mytilene and ‘setting up tents on the town square and going on a hunger strike’, supported by aid groups (Guribye and Mydland, 2018, p. 359). Even then they targeted the refugees and the international NGOs, rather than the Greek state or the EU (Guribye and Mydland, 2018). Here, the conflation between humanitarianism, volunteerism, and activism becomes clear, and problematic, at least in the eyes of the local people who saw their lives as being severely affected by the migration phenomenon on Lesvos.

Aid actors and activists did not seem very aware of this image of themselves, at least that was my impression after a range of interviews and encounters. Or perhaps they did not respond to it or take it seriously, as they had a tendency to perceive of anti‐migration concerns as nationalist, xenophobic, racist, or fascist leanings. Such labels were applied often during my interviews, including those with an international aid worker in her own home,^18^ local activists I met in bars or their offices,^19^ and a journalist who was reporting on these matters.^20^ This is not surprising given the needs and plight of the asylum seekers and the urgent attempts to protect, assist, and engage with the arrivals. Hence, issues concerning reputation or gossip among host communities may not have been such a big issue. Moreover, hesitation to amplify anti‐NGO or anti‐migrant sentiment is a logical institutional reflex by aid groups. Yet, denoting general criticism of asylum and migration in Lesvos as nationalism, xenophobia, racism, or fascism risks pathologising political thinking and sustaining opposition between humanitarians and monstrosity. Positionalities aside, it is important that proponents of the far‐right remain seen as human and not be buried under ‘‐isms’ deemed so extreme as to forego explanation and engagement. Indeed, they can be regarded as ‘rational, fellow human beings with concerns and worries within a society where an extreme style of language and confrontational way of thinking has become “the new normal”’ (Hervik, 2021, p. 94). This also applies to creating opposition between the extreme and the mainstream right. Mondon and Winter (2021) warn that amplifying and ‘hyping’ the presence and significance of such far‐right sentiments and groups may lead to ignoring, distracting from, or legitimising more structural, institutional, or mainstream forms of hate, inequality, and scapegoating. In other words, the far right and its racism ‘develop, thrive, and recede in a wider context, and [this] is shaped by the social, economic and political contexts in which they exist’ (Mondon and Winter, 2021, p. 377). The humanitarian border is such a political context, and by focusing on excesses and extreme rhetoric, the setting in which these politics, discourses, and actions emerged can be overlooked, as can more moderate responses that resort to, develop into, or are picked up by more extreme voices. As such, it is imperative to engage with how aid, care, and assistance are entangled with securitised borders and their violent and discriminatory nature.

## 4 RESONANCE OF CONCERN AND THE FAR RIGHT

An activist in a small town along the coast, slightly north of Mytilene, showed me footage from Facebook and YouTube of how local youths rallied against the arrival of asylum seekers at some time in early 2020. The setting was a small harbour not far from where we were sitting, where a crowd had gathered to prevent the mooring of two boats from Turkey. People were pushing the vessels away while shouting at the asylum seekers, until police intervened. The activist highlighted the escalation in anti‐migration protests on the island, and added that she felt sorry for the protesters, especially the youths. Although she did not condone their actions, she argued that they were victims too. She knew them as they were part of her community, and contended that they were influenced by nationalists and far‐right groups from the island, the mainland, and abroad.^21^

Various other people I met mentioned the influence of far‐right groups. For instance, a solidarian at the Bobiras cafe, an activist meeting point in Mytilene, explained how the *EU–Turkey Statement* coincided with the rise to power of Syriza in 2015.^22^ This leftist party formed a government during the Greek monetary crisis (see below) and ushered in a range of severe austerity measures. Coincidentally, the transit of asylum seekers stopped because of the stalling of implementation of the Statement, just after the political left had come to power. The political right, in response, started to focus on the migration issue, he explained, and mobilised people on social media using entries such as ‘Muslims come to take over’ and ‘migrants are moving to Greece as a human tsunami’. They blamed Syriza for allowing this to happen.^23^

Greece experienced an economic crisis in 2015, and islanders were subjected to currency control measures and cutbacks that impacted their lives significantly. In effect, there was an ‘overlapping crisis’ whereby asylum seekers and citizens were situated in a ‘shared precarity continuum’ (Spathopoulou and Carastathis, 2020, p. 1069). In those early years, aid distribution and the reception of asylum seekers took place in a situation where many islanders themselves experienced economic struggles, if not destitution or fear thereof. As a result of this economic contraction, a shift in individual and local organisational attitudes towards asylum seekers occurred in Greece (Fotaki, 2022). Similarly, Papataxiarchis (2016b, p. 6) noted in 2016 that ‘there is a lot of public resentment against the powerful role of the NGOs, and the camps. are strong indices of the limited powers of the state and municipal authorities. They work as powerful reminders of the current Greek predicament and the “crisis within the crisis” which the islanders experience’.

In this shared precarity continuum, ‘right‐wing groups such as Golden Dawn and Patriotic Movement of Lesvos organized rallies and protests against the presence of asylum seekers and refugees, and at one point physically assaulted and injured volunteers’ (Guribye and Mydland, 2018, p. 358). Protests had happened before the 2020 escalation and confrontations between right‐ and left‐wing activists were a repeated occurrence in Mytilene. There were regular skirmishes and clashes in the Lesvos capital between these groups, often commencing during demonstrations on Sappho Square in the city centre or marches in the streets.^24^

A growing anti‐immigrant sentiment among locals, in sharp contrast to the welcoming attitudes during the arrivals of 2015–16, was used and amplified by conservative politicians and the far right (Vallianatou, 2023). Indicatively, many aid groups, including more established NGOs, had resorted to hiding their institutional affiliations when venturing outside of camps, or going into town. Volunteers were told not to wear T‐shirts with NGO logos in public, and vehicles were anonymised (Jansen, Weishaupt, and Lubberdink, 2020). Local aid initiatives were also threatened, as these were seen as aiding or sustaining asylum seekers' presence on Lesvos. For instance, some hotels refused to house aid groups and their volunteers (Guribye and Mydland, 2018, p. 358). Local businesses that assisted asylum seekers were threatened and, in some cases, attacked by local groups. I visited and interviewed several shop and restaurant owners who worked with asylum seekers who experienced this intimidation. They spoke of a rift in society in and around Mytilene. One such person noted: ‘we have a lot of enemies, more than international NGOs. People are angry; they think we support this bad situation’.^25^ An owner of a fish shop in a small village next to a lake west of Mytilene mentioned that he lost clientele and was threatened by his neighbours for allowing and supporting migrants. It shows a ‘border that runs through crisis, so that which “crisis” you are discursively constructed to be experiencing depends on which side of the border you are on’ (Spathopoulou and Carastathis, 2020, p. 1070). This is interesting when trying to understand the frustration and anger of people.

Greece saw more far‐right violence than any other country in Western Europe in the five years preceding the Lesvos riots, and it was viewed as related to the economic hardship of the country (Jupskås and Fielitz, 2020). The migration question materialised in this setting, and far‐right activists from across Europe supported their Greek brethren in protesting against migration. Lesvos came to be seen as a ‘frontline’ for far‐right groups such as the Identitarian movement (Fallon, 2020). The same media exposure that drew volunteers and aid groups to the island lured political leaders and supporters from Western Europe to protest against asylum reception, with one group referring to itself as ‘Soldiers of the Cross holding the line’ (TRT World, 2020). Activists and volunteers on the island monitored such arrivals, and suspected ‘fascists’ were reported and their whereabouts were shared on social media and in WhatsApp groups.^26^ Just before the clashes broke out in early 2020, two German ‘Nazis’ who arrived at the airport were beaten up by local activists and then ‘thrown out’ (MacGregor, 2020).

The Mediterranean ‘migration crisis’ coincided with the emergence of far‐right political momentum, as nationalists saw themselves as struggling for the survival of European societies that were at risk of growing immigration and multiculturalism (Murray, 2017; Teitelbaum, 2017; Keşkekci and Nissen, 2023). According to this viewpoint, the ‘migration crisis’ and its aftermath in the Mediterranean were playing a key role in debates and conspiracies about ‘the great replacement’: the idea that white European people are being replaced or ‘diluted’ by Black, Arab, and Asian populations (Murray, 2017). In these debates, Mediterranean islands such as Lampedusa and Lesvos, as well as the Canary Islands (a Spanish archipelago off northwest Africa), the Spanish enclaves of Ceuta and Melilla in Morocco, and other areas, are sites where immigration has been framed as eroding European identity, similar to other ‘spectacular borders’ that were surrounded by ‘discourses of invasion’ (Vives, 2017), and where migration was associated with a ‘human tsunami’ (Alexandrakis, 2019). Words like ‘floods’, indicating a sense of hazard, of being besieged, were widely utilised by newspaper and media outlets (Hage, 2016, p. 39). The casting of the Lesvos case as an exemplary example of asylum seekers as a threat to stability and cultural identity in Europe sustained and contributed to an interpretative xenophobic frame (Rucht, 2018, p. 229). The more voice was given to such a threat, linked to local stories about the involvement of aid groups in luring and accommodating asylum seekers, legitimised this interpretative frame, and triggered mobilisation into action.

Yet, to cast the escalation only as a direct result of this far‐right rhetoric is problematic. One evening during the fieldwork I walked around Moria town with an interpreter known in the area to meet with people and talk about the escalation. I spoke with more than 10 individuals, ranging from persons in middle age to a bit older, who had seen the ‘migration crisis’ evolving. With some, we sat on porches, in gardens, and had coffee, and I asked them about their experiences and perceptions of the escalation. With others, we ended up in a bar to discuss the matter further over snacks and Ouzo. These islanders felt that their concerns had been ignored for years, while the asylum situation had escalated over time. There were well aware of the labels that were bestowed on them, such as being racists and nationalists, but they disagreed and argued that they were not racist but critical of how asylum was being handled. They shared an impression that their island was ‘sacrificed’ by the government in Athens, and by the EU in Brussels, Belgium. In different narratives, they asserted that Lesvonians had been hospitable and largely welcomed the asylum seekers from the beginning, but conditions had grown worse over time. ‘We're being destroyed’, one person said. They talked about material damage and fear but also espoused a sentiment of abandonment and neglect by the authorities. This resonates in other resources also, which indicate how local peoples' concerns about migration on the island were obscured or ostracised from the outset (Papataxiarchis, 2016b; Guribye and Mydland, 2018; Vallianatou, 2023). Even after the Moria camp burnt down, later in 2020, government agents promised to assist community members whose properties were damaged or destroyed, but they were never heard from again, as happened before. Indicatively, this obscuring took place while aid groups became the visible enablers of an asylum reception process that had stalled and turned Lesvos into the site of a migration crisis. In relation to the villagers' experiences, however, these aid groups, similar to the government, did nothing to reach out or engage with the host community. In the study interviews held in 2020 and 2021, aid actors confirmed that they had paid little attention to the host community, although some had tried.

Blaming NGOs, volunteers, and activists for the arrival of asylum seekers is shifting the blame, argued a journalist I interviewed in the Pali Agoras cafe—another Solidarian ‘hangout’ in Mytilene, and one that was attacked by angry right‐wing crowds on several occasions, including during the escalation in March 2020.^27^ However, failure to recognise the NGO‐ification of migration management and associated discontent ignores an important dynamic of the humanitarian border process and the role of aid groups and how this was perceived on the island, at least by some. To comprehend the humanitarian border around the Mediterranean region, and perhaps the EU at large, and to consider how it might evolve in the future, effort is required, inter alia, to empathise with the ideas that concerned people have about it, and who may feel disenfranchised and threatened by this theatre of sovereignty.

There is no ready‐made answer to how aid groups and activists can escape the NGO‐ification of migration management along the humanitarian borders they operate in and co‐shape, as well as its impacts and the images left among host communities. And aid groups certainly cannot take on the burden experienced by these communities themselves, as, indeed, broader structural political factors mould the conditions of asylum access and reception. When disenfranchised, frustrated, and angry people feel unheard, they might ultimately fall into the hands of far‐right organisations and forms of discourse that do take their concerns seriously or appear to do so (Limpkin, 2021). They may then mobilise for escalation, or legitimise or stir up violent protest. Aid groups should engage with local communities, even angry ones, and acknowledge feelings of loss, fear, and anxiety among people as something real, rather than spoilers of or threats to humanitarian ideals and practices per se. The desire and aspiration to assist asylum seekers in need and convictions with regard to their entitlement to be received in Europe do not disappear when local concerns are taken seriously, and where possible, addressed, or even advocated for in conjunction with the plight of refugees and asylum seekers. But it also takes a change in mindset to think and communicate beyond labelling people as nationalist, racist, xenophobic, and fascist, and to take concerns seriously as legitimate expressions of local grievance, worry, or interest, regardless of prior political stereotypes, fear, and agendas.

## 5 CONCLUSION

This article has sought to characterise the violent escalation that occurred on Lesvos in early 2020 as intrinsic to the evolution of a particular asylum dynamic. It suggests that a too narrow focus on the extremities and excesses of far‐right rhetoric and mobilisation may obscure more structural aspects of the humanitarian border as a violence‐producing environment. As anti‐asylum protests increased and violence and threats were directed at aid groups, far‐right influences were brought to the fore as instigating such demonstrations. Yet, underlying the targeting of aid groups was a perspective on them, as well as volunteers and activists, as being complicit in the asylum problem on the island, and hence they became targets when the opportunity for rioting arose, and during which people were mobilised.

How aid groups were seen locally, at least by enough people to cause such a violent escalation, and how this relates to the roles and activities of aid groups, is imperative to understanding animosity towards them not as incident, but as intrinsic to such humanitarian border processes. This raises important questions about the nature of humanitarianism and political activism in these border areas, and the extent to which actions inspired by care can also be related to unintended or even anti‐humanitarian effects. The paper argues that in the grey area between concern and protest, neglect of local concerns may grow, and it can be picked up or amplified by political actors that represent opposition to the status quo, that is, far‐right or other radical groups. Greater recognition of the multiplicity of experiences and the challenges of host populations in migration hotspots and along humanitarian borders is vital for sustainable humanitarian governance.

## ETHICS STATEMENT

This paper reports analysis of primary data. Persons from whom data were collected gave their free, prior, and informed consent. Their data have been kept confidential and used anonymously.

## Data Availability

Research data are not shared.^28^

## Endnotes

^1^
The fieldwork and online interviews in 2020 were part of a study by Jansen, Weishaupt, and Lubberdink (2020). This work assessed how local and international actors engage with asylum seekers, host communities, and protest groups that mobilise against each other and the organisations governing and assisting the migration hotspots on the islands of Chios, Lesvos, and Samos in Greece.

^2^
More specifically, the fieldwork in July 2021 consisted of: five interviews with members of local aid groups; six interviews with international volunteers; three interviews with staff of international NGOs; two interviews with business owners; two interviews with local activists; and one interview with a foreign journalist. Two focus‐group discussions and four interviews were held with local community members living in the direct vicinity of the Moria refugee camp. In addition, I have engaged with a wider range of actors, volunteers, and students working in and on Lesvos over the years, including interviews for a previous project (see Jansen, Weishaupt, and Lubberdink, 2020). In certain instances, I refer to such communications without specifying a date and place, as these were part of evolving thoughts and ideas about asylum migration and assistance in the Greek hotspots, based on such encounters.

^3^
For the original *EU–Turkey Statement*, see https://www.consilium.europa.eu/en/press/press-releases/2016/03/18/eu-turkey-statement/ (last accessed on 24 January 2025).

^4^
For an interesting memoir of such a tour, see Heighton (2020).

^5^
Online group interview with four international NGO staff, 24 August 2020.

^6^
Report written by someone with an aid group on the island, titled *Chronic of the Violent Attacks on the Island of Lesvos, Jan–March*. It was shared privately as part of the initial inquiry in 2020, on the condition of anonymity.

^7^

*Chronic of the Violent Attacks on the Island of Lesvos, Jan–March*, p. 2.

^8^

*Chronic of the Violent Attacks on the Island of Lesvos, Jan–March*.

^9^
Interview a local aid worker and activist, Mytilene, 2 July 2021.

^10^
Interview with a foreign journalist, Mytilene, 7 July 2021.

^11^

*Chronic of the Violent Attacks on the Island of Lesvos, Jan–March*.

^12^
Interview with a local woman, Mytilene, 4 July 2024.

^13^
Such impressions are the result of various interactions, such as an online group interview with four international NGO staff members, 24 August 2020, those reported in Jansen, Weishaupt, and Lubberdink (2020), and many of my encounters with NGO personnel and volunteers during my fieldwork in 2021.

^14^
Interview with a local woman, Mytilene, 5 July 2021. Others reflected on these ideas during focus‐group discussions in Moria and conversations in Panagiouda.

^15^

*Chronic of the Violent Attacks on the Island of Lesvos, Jan–March*.

^16^

*Chronic of the Violent Attacks on the Island of Lesvos, Jan–March*, p. 2.

^17^
Group interview with villagers, Moria, 5 July 2021.

^18^
Interview with an international aid worker, Mytilene, 6 July 2021.

^19^
Conversations with two international volunteers working with two different aid groups and two local activists interviewed in Paralia Thermis, 3 July 2021, and Mytilene, 5 July 2021.

^20^
Interview with a foreign journalist, 8 July 2021.

^21^
Interview with a local activist, Paralia Thermis, 3 July 2021.

^22^
Interview with a local activist, Mytilene, 5 July 2021.

^23^
See for a more detailed analysis of anti‐migration mobilisation on social media by far right political actors: https://www.isdglobal.org/wp-content/uploads/2021/07/The-networks-and-narratives-of-anti-migrant-discourse-in-Europe.pdf. Similar experiences were shared in interviews also.

^24^
Three students did fieldwork on Lesvos on humanitarian positioning, volunteer humanitarianism, and the state of exception under my supervision between 2018 and 2021. They experienced these tensions first hand and reflected on them in their projects and in our discussions; see also Strickland (2018).

^25^
Interview with a restaurant and shop owner, Skala Kallonis, 6 July 2021.

^26^
Interview with two volunteers, Mytilene, 3 July 2021.

^27^
Interview with a foreign journalist, Mytilene, 8 July 2021.
